# Agent consumption with the Zeus® in the automated closed circuit anesthesia mode with O_2_/air mixtures

**DOI:** 10.1186/1756-0500-7-469

**Published:** 2014-07-23

**Authors:** Sofie De Cooman, Jan FA Hendrickx, Philip John Peyton, Jean-Luc Demeere, Andre M De Wolf

**Affiliations:** 1 Department of Anesthesiology, Kliniek Sint-Jan, Brussels, Belgium; 2 Department of Anesthesiology/CCM, OLV Hospital, Aalst, Belgium; 3 Department of Anesthesiology, Austin Hospital & University of Melbourne, Melbourne, Australia; 4 Department of Anesthesiology, Northwestern University Medical School, Chicago, Illinois, USA

**Keywords:** Inhaled anesthetics, Equipment, Closed circuit anesthesia

## Abstract

**Background:**

Earlier software versions of the Zeus® (Lübeck, Dräger, Germany) failed to provide true closed circuit anesthesia (CCA) conditions. We examined whether the latest software (SW 4.03 MK 04672–00) achieves this goal.

**Methods:**

In 8 ASA I–III patients, the CCA mode of the Zeus® was used to maintain the inspired O_2_ (F_I_O_2_) and end-expired sevoflurane % (F_Asevo_) at 50 and 1.8%, respectively. The fresh gas flow (FGF) of O_2_ and air and the sevoflurane injection rate (=Vinj_sevo_, mL liquid sevo/h) were videotaped from the control screen and entered offline into a spreadsheet. Cumulative sevoflurane usage during early wash-in (=0-1 min, CD_sevo_0-1), late wash-in (=1-5 min, CD_sevo_1-5), and maintenance (=5-60 min, CD_sevo_5-60) was calculated, and Vinj_sevo_ between 1 and 60 min was compared with published uptake data.

**Results:**

F_Asevo_ reached 1.8% within 101 (23) sec. CD_sevo_0-1 was between 1.24 (0.03) and 3.01(0.25) mL (a range is provided because no absolute Vinj_sevo_ values were displayed once Vinj_sevo_ was > 100 mL/h, which occurred between 15 ± 2 and 46 ± 6 sec). CD_sevo_1-5 was 0.81 (0.37) mL, and CD_sevo_5-60 was 4.63 (0.94) mL. The Vinj_sevo_ pattern between 1 and 60 min matched previously published uptake data. Brief high FGF periods were used to maintain the target F_I_O_2_, and to refill the reservoir bag after external pressure had been applied to the abdomen; subsequent “spikes” wasted 0.08-0.19 mL and 0.14-0.49 mL sevoflurane (1-3% and 3-9% of total agent usage between 1 and 60 min, respectively).

**Conclusion:**

Under the conditions specified, the Zeus® approaches CCA conditions so closely that further reductions in agent usage would have minimal economic significance.

## Background

Closed-circuit anesthesia (CCA) conditions exist when the amount of agent and carrier gas administered match the amounts needed to prime the circle breathing system, taken up by the patient, and lost via leaks. The initial version of the only commercially available automated closed circuit anesthesia (CCA) machine at the time of the study, the Zeus® (Lübeck, Dräger, Germany), failed to reduce agent usage to levels approaching CCA conditions, because FGF usage during the first minutes had been programmed excessively high [1,2]. New software now limits the duration and the pattern of the initial high FGF period and has limited the number of intermittent high FGF flushes during maintenance (intended to reduce unwanted gases like N_2_, compound A, carbon monoxide, methane). We hypothesize this should improve the performance by reducing agent and carrier gas usage to near closed-circuit conditions, and therefore studied the sevoflurane usage with the latest software version, SW 4.03 MK 04672–00, in the automated CCA mode.

## Methods

After obtaining IRB approval (IRB of the Kliniek Sint Jan, Brussels, Belgium; Human studies number OM072 Ref 2012.115) and written informed consent, 8 ASA I –III patients presenting for abdominal or breast surgery were enrolled. The patients’ age, height, and weight were recorded. One hour prior to surgery, 0.5 mg alprazolam p.o. was administered. After preoxygenation with 8 L/min O_2_ by facemask, anesthesia was induced with sufentanil (0.1 mg/kg) and propofol (3 mg/kg). Intubation of the trachea was facilitated by rocuronium (0.5 mg/kg) or cisatracurium (0.1-0.15 mg/kg).

After connecting the endotracheal tube to the anesthesia circuit, ventilation was mechanically controlled with the Zeus® anesthesia machine (software version SW 4.03 MK 04672–00), with tidal volume = 500 mL, respiratory rate = 10/min, and I:E ratio = 1:2. The attending anesthesiologist was allowed to adjust ventilation to maintain normocapnia. Anesthesia was maintained with sevoflurane in O_2_/air using the automated CCA mode. Time zero was the time at which the target F_I_O_2_ was set at 50% and the target end-expired sevoflurane concentration (F_A_sevo) at 1.8%. From this point on, the machine automatically adjusted the O_2_ and air FGF as well as the sevoflurane liquid injection rate (Vinj_sevo_) to attain and maintain the specified targets. Additional sufentanil and rocuronium or cisatracurium administration were left at the discretion of the attending anesthesiologist. The study arbitrarily lasted 60 min.

Because we could not obtain software to download the data with a resolution sufficient for the purposes of this study (at least every second), the monitor screen was video-recorded. The values of the following parameters were entered offline every second into a spreadsheet (amounting to 3600 entries per parameter per patient): (1) time; (2) the O_2_ and air FGF and Vinj_sevo_; and (3) the resulting inspired and end-expired O_2_, CO_2_, and sevoflurane concentrations (F_I_O_2_, F_A_O_2_, F_I_CO_2_, F_A_CO_2_, F_Isevo_, and F_Asevo_, respectively).

Cumulative sevoflurane consumption was obtained by integrating the area under the curve of Vinj_sevo_. The Vinj_sevo_ value is displayed on the Zeus screen. This value is the average of multiple, very precisely dosed liquid “pulses” injected per second from the so-called DIVA cassette, basically a fuel-injector from a car engine that injects liquid agent with precise dosing volume of 3 to 50 microL (Wilfried Buschke, Dräger, personal communication).

To assess how closed the Zeus® worked, we compared the Vinj_sevo_ pattern with previously published sevoflurane uptake data derived from several different sources: closed circuit liquid injection [3,4], indirect calorimetry (gas balances within the circuit) [5,6], and the reverse Fick method [5,6]. A proportional correction was applied to account for differences in F_A_sevo. Patient demographics, cumulative agent usage, and times are presented as mean ± standard deviation. Because other data (FGF, Vinjsevo, F_A_sevo) are not normally distributed, they are presented as median and quartiles.

## Results

**Figure 1:**
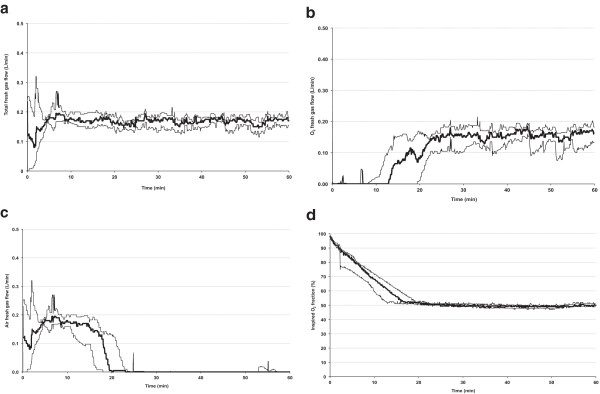
**Carrier gas characteristics.** Total fresh gas flow remained below 200 mL/min **(a)**, but the composition of the fresh gas changed over time: air fresh gas flow **(b)** decreased, while O_2_ fresh gas flow **(c)** increased. The inspired O_2_ concentration progressively decreased towards the target of 50% **(d)**. Thick lines = median, thin lines = quartiles.

Patient age, height, and weight were 58 ± 13 years, 164 ± 9 cm, and 77 ± 15 kg. Median total FGF (Figure 1a) remained between 150 and 200 mL/min throughout the procedure, with the composition changing progressively from air to O_2_ (Figure 1b,c) as the F_I_O_2_ decreased slowly towards the target 50% (Figure 1d). High FGF bursts were few (Table 1), and were mainly used to increase F_I_O_2_ to its target or to fill the breathing bag after pressure had been exerted on the abdomen or thorax by the surgical team that resulted in loss of gas from the system.

**Table 1:** Overview of intermittent high fresh gas episodes and ensuing sevoflurane spikes

		**High FGF episodes**					**Sevoflurane spikes**					
**Etiology**	**Message**	**Time**	**Av FGF**	**Composition**	**Duration**	**Volume wasted**	**V**_**sevo **_**prior to spike**	**V**_**sevo **_**during spike**	**Duration**	**Sevo waste**	**% of average usage 1–60 min (5.44 mL)**	**Pt**
		**min**	**L/min**	**% O**_**2**_	**sec**	**L**	**mL/h**	**mL/h**	**sec**	**mL**		**#**
F_A_sevo overshoot +	Autoflush	2	6.4	21	36	3.86	part of washin after overshoot,			N/A		1
CO_2_ sampling issue	Autoflush	7	2.1	100	31	1.10	followed by sampling issue			N/A		1
During calibration	Calibration	27	2.0	33	19	0.65		no change		N/A		2
Pressure on abdomen	Bag filling	14	14.0	38	7	1.64	7	84-198	7	0.15-0.39*	3-7	5
	Bag filling	20	13.8	100	7	1.61	8	79-165	7	0.14-0.32 *	3-6	4
	Bag filling	25	13.3	99	6	1.33	6	58-131	11	0.16-0.40*	3-7	5
	Bag filling	25	13.2	21	6	1.32	4	72-172	8	0.15-0.38*	3-7	2
	Bag filling	33	16.6	100	6	1.66	4	89	6	0.14	3	3
	Bag filling	34	16.9	98	6	1.69	4	100-300	6	0.16-0.49*	3-9	3
F_I_O_2_ < 50%	Autoflush	45	1.9	100	27	0.87	5	6	209	0.08	2	6
	Autoflush	51	2.3	100	26	0.98	7	21	20	0.08	1	4
	Autoflush	52	1.9	100	33	1.07	5	7	311	0.19	3	7
	Autoflush	55	1.7	100	37	1.04	2	4	254	0.16	3	1
Pressure on abdomen + F_I_O_2_ < 50%	Bag filling, then autoflush	36	3.1	60	32	1.68	4	13	60	0.15	3	3

F_A_sevo = end-expired sevoflurane %; FGF = fresh gas flow; Av = average; Vsevo = sevoflurane usage; Pt = patient; N/A = not applicable.

*Sevoflurane injection rate at least temporarily > 100 mL/h, thus a range is given. See text for details.

**Figure 2:**
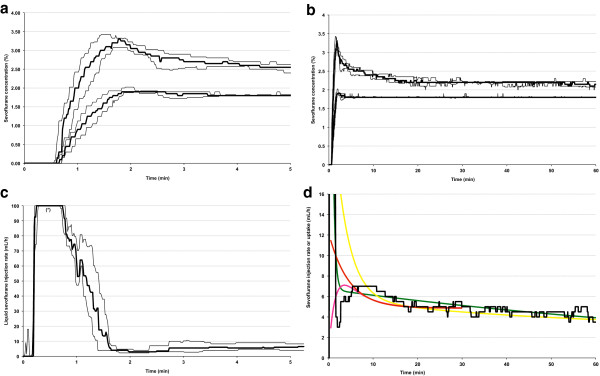
**Inspired and end-expired sevoflurane concentrations during wash-in (a) and maintenance (b) (upper and lower line, thick line = mean, thin lines = standard deviation), and corresponding liquid sevoflurane injection rate (mL/h) (c and d, respectively).** During the first 5 minutes **(c)**, the liquid sevoflurane injection rate (mL/h) could not be accurately measured between 15 ± 2 and 46 ± 6 sec because Vinj_sevo_ was > 100 mL/h (see text for details). The sevoflurane injection rates match those of previously published sevoflurane uptake data derived from several different sources **(d)**: closed circuit liquid injection (yellow line = Lockwood data, green line = Hendrickx data) [3,4], indirect calorimetry (gas balances within the circuit = red line) [5,6], and the reverse Fick method (pink line) [5,6]. Thick lines = median, thin lines = quartiles.

F_Asevo_ reached 1.8% within 101 ± 23 sec (Figure 2a) and was maintained within 0.1% thereafter (Figure 2b). After the first minute, the Vinj_sevo_ pattern matched previously published uptake data [3-6] (Figure 2c). Vinj_sevo_ “spikes” followed the high FGF episodes mentioned above, and wasted 0.08-0.19 mL and 0.14-0.49 mL liquid sevoflurane when used to increase F_I_O_2_ and after external pressure had been applied to the abdomen, constituting 1-3% and 3-9% of total agent usage between 1 and 60 min, respectively (Table 1).

Cumulative liquid sevoflurane usage was 0.81 ± 0.37 mL between 1–5 min, and 4.63 ± 0.94 mL between 5–60 min. The cumulative sevoflurane usage between 0 and 1 min could not be accurately measured because Vinj_sevo_ was > 100 mL/h between 15 ± 2 and 46 ± 6 sec (Figure 2d); under these circumstances, the Zeus ceases to display values and only displays a message “Vinj_sevo_ > 100 mL/h”. Still, cumulative agent usage can be reported as a range because the maximum Vinj_sevo_ = 300 mL/h, thus the injection rate during these “blackout” episodes has to lie between 100 and 300 mL/h. For example, for a 12 sec blackout period, the cumulative sevoflurane amount ranges between 0.33 to 1 mL liquid. According to these injector limits, cumulative sevoflurane usage between 0 and 1 min was between 1.24 ± 0.03 and 3.01 ± 0.25 mL.

## Discussion

While older studies found that with the older algorithms the Zeus® could not reduce agent usage to the amounts needed to prime the system and to replace the amounts taken up by the patient [2], our current data suggest that the newest algorithms applied by the Zeus® have succeeded in reducing agent usage to just 4–12% above CCA conditions [3-5]. Besides software improvements, other factors may account for this.

First, the algorithms steering the FGF and agent injector during wash-in have been optimized. The Zeus® manages FGF and agent administration to attain and maintain the initial target F_A_ of the agent and carrier gases in such a manner that an acceptable trade-off is made between the speed of reaching the targets (F_A_O_2_, F_A_N_2_O, and F_A_ agent) and usage of carrier gas and agent. Requests for a higher F_I_O_2_ or lower F_A_ agent are considered high priority, necessitating a *rapid* “step” change and thus a high FGF, which comes at the price of increased agent usage. The response to a request for a lower F_I_O_2_ may be allowed more time. Exceptions may include the use of laser in the airway, or the care of neonates where a fast reduction in F_I_O_2_ may be required. This can be accomplished by the use of a fast flush button pre-programmed with a FGF according to the user’s preference, but the usage of any concomitantly administered agent will increase. A request for a higher F_A_ agent does not require a high FGF because agent administration and FGF are mechanically uncoupled: agent is injected by the DIVA cassette, and a blower ensures rapid mixing in the circuit. The high FGFs programmed into the early software during initial wash-in have been rewritten: F_I_O_2_ is allowed to drift gradually towards the target F_I_O_2_ if the initial F_I_O_2_ is higher than the target F_I_O_2_. Figure 1 illustrates that the average total FGF during the first 5 min varies within a small marge of 150–210 mL/min.

Second, N_2_O used in previous studies may have increased agent usage compared to N_2_ (used in this study). If N_2_O is used, high initial FGF are required to achieve sufficiently high concentrations of N_2_O in a fairly short time period. Therefore, it is more difficult to maintain CCA from the very beginning of the anesthetic, and agent usage will initially be higher compared to when O_2_/N_2_ is used. However, (1) F_A_N_2_O may be allowed to rise more gradually, and (2) the effect of N_2_O on agent consumption is bimodal: after a while, total agent usage doses become lower when N_2_O is used because a lower agent concentration can be used [7].

Third, few high FGF bursts (flushing) were used during the maintenance phase (Table 1). What purpose do these high FGF bursts serve, to what extent do they increase agent usage, why were they infrequent in our study, and can they be further reduced or eliminated all together? Earlier software versions commanded “routine” intermittent flushing of the breathing system to eliminate unwanted gases like CO, compound A, or methane. However, their clinical relevance has become questionable (with KOH free CO_2_ absorbents) or entirely irrelevant (with KOH and NaOH free CO_2_ absorbents). These “routine” bursts have therefore been eliminated, but the concerned anesthesiologist can activate the flush button.

Flushing is also initiated after a certain N_2_ threshold has been exceeded when N_2_O/O_2_ is used, or more precisely after the 10% “balance gas” threshold has been exceeded (N_2_ continues to be released from slowly equilibrating tissues and thus slowly accumulates in the circuit). The balance gas is calculated as follows: 100% - (F_I_N_2_O + F_I_O_2_ + F_A_agent + 7 vol% H_2_O). Because we used O_2_/air as the carrier gas, and because the F_I_O_2_ was allowed to gradually drift down towards the target F_I_O_2_, very few high FGF bursts were needed (n = 5, total wasted carrier gas volume 1.1 ± 0.3 L) (Table 1). But even with the use of O_2_/air mixtures, the continued release of N_2_ may cause the F_I_O_2_ to eventually drop below its target, initiating a brief O_2_ flush, which occurred in 4 patients after 45 min (Table 1).

Six FGF bursts were also prompted by a decrease of the pressure in the breathing bag at end-expiration caused by pressure exerted on the abdomen or thorax by the surgical team; these were short-lived (6.3 ± 0.5 sec) and wasted 1.5 ± 0.2 L of circuit gas. The bag is part of the FGF uncoupling system; if the pressure in the bag is not slightly positive at the end of expiration, the system perceives this as lack of sufficient fresh gas, and the FGF is increased. Three other bursts occurred after gas sampling line kinking (for safety reasons no rebreathing is allowed without 2 properly functioning gas analyzers), F_A_sevo overshoot (a maximum increase of 15% above target is allowed), or post-calibration (even though this did not happen after other calibration periods).

FGF bursts are also used to rapidly attain new targets during the maintenance phase. Demand for a higher F_I_O_2_ or lower F_A_ agent in particular are considered high priority requests, necessitating a *rapid* “step” change and thus a high FGF period, which does come at the price of increased agent usage. The response to a demand for a lower F_I_O_2_ is allowed more time (see above). A demand for a higher F_A_ agent does not require a high FGF because agent administration does not depend on FGF (it is injected by the DIVA cassette, and a blower ensures rapid mixing in the circuit). Because we did not change the O_2_ and F_A_sevo targets, the number of FGF bursts and thus carrier gas and agent waste were minimized.

We believe our results allow us to conclude that the Zeus decreases agent usage to very-near CCA conditions. During the first minute, the exact amount of liquid sevoflurane usage is unkown, but from the injector limits we can deduct it is between 1.24 ± 0.03 and 3.01 ± 0.25 mL. Future studies might consider weighing the DIVA cassette or determine the amount of agent exhausted towards the scavenging system. Our preliminary data indicate that sevoflurane waste from the exhaust valve under identical study conditions is 0.014 ± 0.004 mL (range 0 – 0.018 mL) liquid sevoflurane during the first 5 min. The first few minutes of agent delivery are crucial to help minimize agent waste. Comparison of usage data of the first few minutes between studies is complicated by differences in circle system configurations and the rate of rise of the agent concentration. During maintenance, the current software version of the Zeus® reduces total FGF and O_2_ FGF in particular to previously published uptake rates [8]. But the number of these high FGF episodes and their effect on concomitant agent usage has become so small that the excess waste has become negligible vis-à-vis total sevoflurane usage - further reductions would be economically and environmentally insignificant, and technically difficult to achieve.

There are several implications of our findings. First, technology has evolved up to a point where potent inhaled anesthetic agents can be administered with almost no waste. Still, the “purist” can keep agent usage low by minimizing target changes and by accepting that targets be reached gradually [9], principles that apply to any CCA technique. Second, considering that most anesthesiologists still use a maintenance FGF of 1.5 – 2 L/min when using a conventional anesthesia machine [10,11], automating CCA to maintain F_A_sevo at 1.8% in O_2_/air will reduce agent usage during the maintenance phase (0 – 55 min) alone by 367%, from 17.0 mL [12] to 4.63 mL (current study). Yet how this will translate into costs savings is complex, and requires considerable detailed information. For example, one study that compared the costs of the Zeus with the Primus failed to accurately compare the F_A_ agent and the number of F_A_ agent changes, the initial FGFs, and the O_2_ concentrations, precluding any meaningful conclusions to be made from these results [13]. Another example is the imposed use of high FGF when sevoflurane is used due to concerns for Compound A or CO formation: although the newer CO_2_ absorbents do not produce these substances, the increased cost of the more expensive and less efficient KOH and NaOH free CO_2_ absorbent will still be outweighed by the savings made by using less sevoflurane when using an automated CCA machine [14]. Finally, the inhaled anesthetic drugs released during the approximately 200 million anesthetic procedures performed each year globally have a climate impact that is approximately 0.01% of that of the CO_2_ released from global fossil fuel combustion [15]. Technology now enables us to even reduce this by another order of magnitude, making our contribution to ozone layer destruction and green house effect exceedingly small.

## Conclusions

Under the conditions specified in this study, software version SW 4.03 MK 04672–00 has made the automated CCA mode of the Zeus® approach CCA conditions except for brief functional high FGF episodes that result in waste of 3-9% per episode of the total amount of agent administered. Strictly speaking, CCA conditions still were not met 100% of the time, but even under the best of conditions, there always will be some need for intermittent high FGF bursts. It is unlikely this very small amount of waste can be further reduced, and it is likely that any further reduction would be irrelevant. We conclude that the Zeus approaches CCA conditions.

## Abbreviations

CCA: Closed circuit anesthesia; FGF: Fresh gas flow; F_I_O_2_: Inspired O_2_ concentration; F_A_O_2_: End-expired O_2_ concentration; F_I_CO_2_: Inspired F_I_CO_2_ concentration; F_A_CO_2_: End-expired CO_2_ concentration; F_Isevo_: Inspired sevoflurane concentration; F_Asevo_: End-expired sevoflurane concentration; Vinj_sevo_: Sevoflurane injection rate

## Competing interests

The only author who has competing interests is Dr. Hendrickx.

In the past five years, Dr. Hendrickx has received lectures fees, travel reimbursements, and/or research equipment from AbbVie, Baxter, Draeger, GE, Heinen und Lowenstein, Maquet, MEDEC. None of the authors has any other financial or non-financial competing interests.

## Authors’ contributions

SDC and JH conceived the idea; SDC collected and processed the data; all authors contributed to data review, analysis, manuscript preparation, and all gave their final approval.
